# Effects of Unbalance Identification Locations on Transient Dynamic Balancing Without Trial Weights Performance of Power Turbine Rotor

**DOI:** 10.3390/s25237242

**Published:** 2025-11-27

**Authors:** Jiepeng Zhao, Yongfeng Yang, Wangqun Deng, Shibo Zhao, Chao Fu, Xingmin Ren, Zhihua Nie

**Affiliations:** 1 Institute of Vibration Engineering, Northwestern Polytechnical University, Xi’an 710072, China; 2021100200@mail.nwpu.edu.cn (J.Z.); fuchao@nwpu.edu.cn (C.F.);; 2 AECC Hunan Aviation Powerplant Research Institute, Zhuzhou 412002, China; 3 Key Laboratory of Aero-Engine Vibration Technology, Aero Engine Corporation of China, Zhuzhou 412002, China; 4 Beijing Institute of Astronautical Systems Engineering, Beijing 100076, China

**Keywords:** transient balancing, power turbine rotor, filtering principle, unbalance identification, different position

## Abstract

**Highlights:**

The main findings of this paper are summarized as follows:

- First, a transient dynamic balancing method without trial weights was developed for a specific type of power turbine rotor. Based on modal balancing theory, this method identifies the rotor unbalance by calculating the unbalance excitation force.
- Second, the applicability of unbalance identification at different axial correction mass positions was systematically analyzed for the investigated rotor model.

The implications of the main findings are generalized as follows:

- First, the proposed transient dynamic balancing method requires only a single rotor startup and identifies the rotor’s unbalance without adding any trial weights, which significantly improves balancing efficiency.
- Second, the research on the applicability of unbalance identification across various axial correction mass positions on an actual rotor model significantly improves the efficiency of on-site dynamic balancing operations.

**Abstract:**

This study proposes a dynamic balancing method without trial weights for power turbine rotors and investigates how the axial location chosen for unbalance identification affects the balancing performance. A finite element model of the power turbine rotor system was established to compute transient vibration responses and principal modes. Both continuous and isolated unbalances are employed to identify unbalanced excitation forces, enabling the determination of unbalance parameters. Furthermore, variations in identification accuracy across four designated axial positions on the rotor were analyzed. Simulations and experiments conducted on boss 2 and boss 3 confirmed the method’s efficacy: the maximum vibration amplitudes were reduced by 70.48% and 45.81% for boss 2, and by 64.48% and 61.00% for boss 3, respectively. These results verify the effectiveness of the proposed method. The unbalance parameters identified from simulations exhibited errors within ±6°, ±0.12 g, and ±0.15 × 10^−4^ m, while experimental errors remained within ±5°, ±0.11 g, and ±0.10 × 10^−4^ m, demonstrating high accuracy and reliability. Notably, this method improves balancing efficiency by requiring only a single startup and facilitates vibration data acquisition in confined spaces.

## 1. Introduction

Rotor dynamics is an interdisciplinary field focused on the dynamic behaviors of rotors in rotating machinery, including their components and structural systems. Its research scope encompasses vibration analysis [1,2,3], dynamic response [4,5,6], stability assessment [7,8,9], reliability engineering [10,11,12], condition monitoring [13,14,15], fault diagnosis [16,17,18], and vibration control [19,20,21], among others. Given that excessive vibration can lead to rotor system failure and significant safety risks, it has attracted substantial research attention across these domains. With rotor systems now rapidly advancing toward ultra-slender and ultra-high-speed designs, vibration issues must be properly addressed to ensure the safe and stable operation of engines. Since rotor unbalance represents the primary source of vibrations, research into rotor unbalance identification has become increasingly imperative.

With current technology, it is infeasible to completely eliminate unbalance. It is therefore essential to reduce rotor unbalance to ensure that vibration amplitudes in rotating machinery remain within safe thresholds. Additionally, rotor unbalance can induce additional failures, such as excessive noise [22,23,24] and bearing wear [25,26]. An aero-engine is a highly complex and sophisticated thermal machine, serving as the powerplant that provides thrust for aircraft. By burning fuel to generate high-temperature, high-pressure gas, it drives turbines to rotate or produces reaction force through direct jetting, thereby propelling the aircraft forward. It is often referred to as the heart of an aircraft. Enhancing rotor balancing efficiency can effectively mitigate these failures, thereby improving the stability and reliability of aero-engines. Consequently, dynamic balancing of flexible rotors has become an indispensable step in the design and manufacturing process of aero-engines.

Given the complex structure of aero-engine power turbine rotors, research on such rotors has advanced steadily. Nie [27] considered mass loss and component temperature, and proposed a new method to precisely control the blade fracture rotational speed via precisely prefabricated cracks at the blade root. Nie and Wang [28,29] investigated the influence of oil film radial clearances on the dynamic characteristics of a variable-speed rotor system, and then introduced the principles of equivalent rotor dynamics similarity design. Shao [30] studied the nonlinear dynamic characteristics of a power turbine rotor system with a branching structure, and analyzed the influence of oil-film clearance, length, and viscosity on vibration responses. Yue [31] incorporated the numerical method with modal ratio coefficients among measurement points and the triple phase method, and proposed a new modal balancing process for assessing residual unbalance from different equilibrium planes of a complex flexible rotor system, which was applied to the power turbine rotor. Jia [32] developed a new method to determine the optimal installation position of two-stage turbine disks on the power turbine rotor through vibration optimization design, and deduced the transient response of bearing acceleration and disk amplitude. Nan [33] investigated the influence of rotational speed, misalignment angle, and rub-impact clearance on the dynamics of the power turbine rotor system, and showed that increasing the rub-impact clearance can improve system stability. Cao [34] analyzed the vibration response of a turbine engine’s cantilever rotor under different unbalanced phases and their combinations, and studied the effect of different combinations of unbalanced phase differences on unbalance and dynamic balancing.

In engineering practice, unbalance caused by rotor manufacturing errors can induce severe vibrations in rotor systems, compromising the stability and service life of engines. Consequently, the detection and suppression of rotor unbalance have received significant attention from researchers. Zhu [35] proposed a Pre-Adaptive Transfer Learning method that performed sample reconstruction based on frequency domain correlation analysis, greatly improving the accuracy and generalization of unbalanced position identification. Smolík [36] used a multi-body dynamics formalism to describe the inertia force on the rotor, investigated the interaction between static and dynamic unbalance, and provided an analytical relationship depicting how the balancing procedure influences the products of inertia. Hu [37] introduced CEEMDAN to decompose the initial unbalanced signal, analyzed the feature extraction of dynamic unbalanced signals using this method, and successfully verified its advantages in dynamic balancing machine detection. Zhang [38] first introduced the principle of signal purification to acquire pure unbalance information, then proposed an SP-based suppression method for unbalanced vibration in rotors with multiple 1X faults to reduce rotor unbalance. Li [39] used cross-correlation analysis, accurately identifying the magnitude and phase of the unbalanced signal by extracting radial vibration of the blade tip at different speeds and calculating them in the time domain. Zhang [40] introduced the concept of combined unbalanced phase difference, constructed the influence coefficient matrix, and proposed a least squares influence coefficient method based on the Nutcracker optimization algorithm to investigate the effect of combined unbalanced phase difference on high-speed dynamic balancing. Liu [41] presented a method for unbalanced vibration feature extraction based on all-phase fast Fourier transform and developed a micro-motor rotor unbalance test system. Wu [42] proposed a tacholess order tracking method based on the STFTSC algorithm (combining short-time Fourier transform and seam carving algorithm) to precisely identify rotor unbalance. Sun [43] proposed a method for identifying unbalance parameters based on time-domain response, integrating a matrix equation and spectral correction technique (SCT). This method solves the matrix equation obtained from the state space equations of the reduced-order model and uses SCT to process the unbalanced force generated by vibration response. Chen [44] proposed a new assembly datum for unbalance optimization, aimed at incorporating the alignment process and distribution of screw holes in adjacent rotors. A genetic algorithm was used to optimize unbalance levels, and the corresponding optimal assembly orientations of rotors at different stages were accurately identified.

As rotating machinery finds increasingly widespread applications, research into the performance of rotor systems has garnered growing attention. In the field of dynamic balancing in particular, researchers have proposed various improved methods. Jiang [45] put forward a multi-strategy improved sparrow search algorithm that minimizes the sum of squared residual vibrations and the maximum residual vibration at each measurement point. Quinz [46] used the Numerical Assembly Technique for the in-field balancing of warped rotors with flexible behavior, greatly improving the efficiency of on-site balancing. Zheng [47] employed the equivalence principle of magnetic forces to solve for the correction weights in the double-plane correction method for magnetically levitated rotors, and adopted a secondary correction method by revising the conversion coefficient matrix obtained from online identification procedures. Wang [48] proposed an improved algorithm that applies Nonlinear Normal Modes to the modal balancing procedure, thereby enhancing the balancing efficiency of rotor systems with nonlinear mechanical elements. Zhong [49] presented a balancing method without trial weights using an unsupervised deep Lagrangian network, and applied parameter-sharing mechanisms in the baseline design or Lagrangian layer to identify unbalanced forces without labeled data. Yang [50] adopted an optimization method and inverse identification approach to minimize the objective function, then used a genetic algorithm to solve for an optimal balancing scheme. In this scheme, the optimization process, which exists within a global multi-objective optimization framework, is transformed into single-objective and dual-objective optimization. Li [51] optimized the rotor balancing strategy through sensitivity analysis of mode shapes, obtained orthogonal trial masses based on the orthogonality of each vibration mode, and calculated correction masses using the influence coefficients of the trial masses. Quinz [52] computed the generalized unbalance for each eigenvalue by comparing the displacement between simulation and measurement, then calculated orthogonal correction masses by amending the generalized unbalances according to modal theory. Zhao [53] designed a dynamic balancing testing system that integrates a transient characteristic-based balancing method and the influence coefficient method, greatly simplifying the operational steps of on-site dynamic balancing.

This paper proposes a method for identifying unbalanced forces based on the modal balancing theory for a power turbine rotor, with which unbalanced parameters such as azimuth, eccentricity, and magnitude are identified. A balance boss is a dedicated annular or cylindrical structure designed on a power turbine rotor. Its essential function is to correct the rotor’s mass unbalance by adding or removing counterweights at specific angular positions on the boss, thereby reducing vibration during rotation. Subsequently, the differences in unbalance measurements at boss 1, boss 2, boss 3, and the first-stage power turbine disk of the power turbine rotor were investigated through simulations and experiments. Section 2 introduces the theory of unbalanced force identification based on the modal balancing method, along with the method for identifying unbalanced parameters, and additional content related to order analysis is also included. Numerical simulations and dynamic balancing experiments on the power turbine rotor are presented in Section 3 and Section 4, respectively. Finally, Section 5 and Section 6 summarizes the key findings and draws conclusions regarding the research outcomes.

## 2. Methodology for Identifying Unbalance

### 2.1. Methodology for Recognizing Unbalance Parameters

This section introduces the principle of the proposed method. Based on modal balancing theory, the method calculates the unbalance excitation force by synthesizing both continuous and isolated unbalance models of the rotor system, thereby enabling the identification of the inherent unbalance. A key advantage of this approach is that it eliminates the need for trial weights and constant-speed balancing, achieving unbalance identification in a single startup procedure. These attributes collectively lead to substantially improved balancing efficiency and reduced costs for power turbine rotors.

According to the modal balancing principle, an external force distributed in accordance with a specific mode shape will excite vibrations exclusively in that mode. This force alters the amplitude of the corresponding modal component without affecting other modes, a consequence of the orthogonality of mode shapes. This relationship is expressed as follows:

(1) $$\left.\begin{array}{l} \int_{0}^{l}m(z)\phi_{i}(z)\phi_{j}(z)=0,i\neq j\\ \int_{0}^{l}m(z)\phi_{i}(z)\phi_{j}(z)=S_{n},i=j \end{array}\right\},(i,j=1,2,\ldots ,n)$$

where $\phi_{n}(z)$ and $S_{n}$ denote the *n*th principal mode and the modal mass of the *n*th mode, respectively. m(z) represents the continuous mass along the *z*-axis, which is assumed to be in the axial direction. *l* is the total length of the shaft.

The dynamic balancing condition for a flexible rotor is that the force and moment generated by the set of balancing weights added to the rotor must be zero. Therefore, when balancing a flexible rotor without considering damping, the following balancing equations must be satisfied:

(2) $$\left.\begin{array}{l} \int_{0}^{l}P(z)dz+\sum_{s=1}^{k}P_{s}=0\\ \int_{0}^{l}P(z)zdz+\sum_{s=1}^{k}P_{s}z_{s}=0\\ \int_{0}^{l}P(z)\phi_{n}(z)dz+\sum_{s=1}^{k}P_{s}\phi_{n}(z_{s})=0 \end{array}\right\}$$

where P(z) is the distribution function of rotor unbalance, and $P_{s}$ denotes the unbalance equivalent of the *s*th-order mode shape. In Equation (2), the first and second expressions represent the rigid balancing conditions, while the third corresponds to the flexible balancing condition. The relationship between P(z) and $P_{s}$ can be expressed by the third equation of Equation (2).

For the selection of the number of balancing planes, there are two methods: the *N*-plane method and the *N* + 2-plane method. To balance the *N*th-order mode shape, at least *N* balancing planes are required, which is generally referred to as the *N*-plane method. When balancing the *N*th-order mode shape using *N* balancing planes, the rigid mode components in the bearing dynamic reactions cannot typically be completely eliminated. To eliminate the bearing dynamic reactions, two additional balancing planes must be added, a technique commonly known as the *N* + 2-plane method. In practical applications, the choice between the *N*-plane method and the *N* + 2-plane method has been thoroughly reviewed by Kellenberger [54]. This paper identifies the unbalance of the power turbine rotor based on the *N*-plane method.

In rotor systems, unbalanced excitation forces originate from rotor unbalance. These forces consist of distributed and concentrated unbalanced forces, namely continuous and isolated unbalanced forces. According to modal balancing theory, the continuous unbalance of a rotor system can be converted into an equivalent set of a finite number of isolated unbalances. By considering only the effects of the first *N*-order modes of the rotor system, the relevant results can be derived from

(3) $$\left\{\begin{array}{ll} \phi_{1}(z_{1})U_{1}+\phi_{1}(z_{2})U_{2}+\cdots +\phi_{1}(z_{k}) & U_{k}=-\sum_{p=1}^{M}\phi_{1}(z_{p})U_{p}-q_{1}S_{1}\\ \phi_{2}(z_{1})U_{1}+\phi_{2}(z_{2})U_{2}+\cdots +\phi_{2}(z_{k}) & U_{k}=-\sum_{p=1}^{M}\phi_{2}(z_{p})U_{p}-q_{2}S_{2}\\ & \vdots \\ \phi_{n}(z_{1})U_{1}+\phi_{n}(z_{2})U_{2}+\cdots +\phi_{n}(z_{k}) & U_{k}=-\sum_{p=1}^{M}\phi_{n}(z_{p})U_{p}-q_{n}S_{n}\\ & \vdots \\ \phi_{N}(z_{1})U_{1}+\phi_{N}(z_{2})U_{2}+\cdots +\phi_{N}(z_{k}) & U_{k}=-\sum_{p=1}^{M}\phi_{N}(z_{p})U_{p}-q_{N}S_{N} \end{array}\right.$$

where $U_{K}$ denotes the isolated unbalance at point *K*, $U_{p}$ denotes the continuous unbalance of the rotor system, and $q_{n}$ represents the *n*th-order modal component of the unbalanced distribution. The value of $U_{K}$ is obtained when *K* = *N*.

As is well known, the distributed unbalanced forces of the rotor exhibit a uniform distribution along the axial direction. Thus, the distributed unbalanced force can be expanded in terms of the principal mode shapes, enabling the derivation of the continuous unbalance of the rotor system. By combining the continuous unbalance with the isolated unbalance, the unbalanced excitation force of the rotor system can be obtained, which is expressed as

(4) $$Q(t)=\omega^{2}(t)\sum_{n}q_{n}m(z)\phi_{n}(z)+\omega^{2}(t)\sum_{K=1}^{K}U_{K},n=1,2,\ldots ,N$$

where ω(t) denotes the angular velocity of the power turbine rotor.

Considering the unbalanced excitation forces in the *x*-direction, the excitation forces can be expressed as

(5) $$Q_{x}(t)={\left[Q_{x1}(t),Q_{x2}(t),\cdots ,Q_{xr}(t),\cdots ,Q_{xN}(t)\right]}^{T},r=1,2,\cdots ,N$$

The excitation force vectors, which contain the unbalance parameters of the disks, are given by:

(6) $$Q_{xr}=m_{r}e_{r}\sqrt{\left(\omega^{4}(t)+a^{2}(t)\right)}\cos[\alpha (t)+\theta_{ur}]$$

where $m_{r}$, $e_{r}$ and $\theta_{ur}$ denote the mass, eccentricity, and initial unbalance azimuth of the *r*-th disk, respectively. a(t) is the angular acceleration of the power turbine rotor, which can be computed from the key phase signal. α(t) represents the phase term, expressed as $\alpha (t)=\kappa (t)+\eta_{x}(t)$. κ(t) is the rotational angle of the disk, and $\eta_{x}(t)$ can be expressed as

(7) $$\eta_{x}(t)=\arctan\left(-\frac{a(t)}{\omega^{2}(t)}\right),\eta_{x}(t)\in \left(-\frac{\pi }{2},0\right)$$

From Equation (4), the unbalanced excitation force at a specific point on the power turbine rotor, where the correction mass needs to be added, is identified. Subsequently, all minimum points of the unbalanced excitation force are selected as feature points for calculating the unbalance. The unbalance at the point where the correction mass is to be added (including azimuth, eccentricity, and mass) can be identified using Equations (6) and (7), and dynamic balancing is achieved by adding an equal correction mass in the opposite direction.

### 2.2. The Principle of the Order Analysis

The order analysis method transforms non-stationary time-domain signals into stationary signals in the angular domain. This approach effectively mitigates various mechanical excitations and inherent system random errors, which are often difficult to suppress in the time, frequency, or time-frequency domains.

Order analysis is fundamentally reliant on rotational speed signals. An order, defined as the number of vibration events per rotation cycle, provides an ideal measure for characterizing speed-dependent vibrations. The relationship between order, rotational speed, and frequency is quantified by

(8) $$O=\frac{60f}{n_{rs}}$$

where *O* denotes order, *f* denotes frequency, and *n_rs_* denotes rotating speed.

The core of order analysis is the acquisition of a noise signal through equiangular sampling, also referred to as order tracking. This process requires the sampling trigger interval to correspond to the time taken for the engine to rotate by a fixed angle. To maintain a constant number of samples per revolution despite speed variations, the sampling rate must be synchronized with the engine’s rotational speed. The procedure involves two primary stages. First, equitemporal sampling is performed, where the original noise signal and a rotational speed pulse signal are synchronously captured at a fixed rate via separate channels. Second, interpolation resampling is conducted: the rotational speed is estimated from the pulse sequence to compute the exact time instances for equiangular sampling. The synchronously sampled noise signal is then interpolated at these computed time points, producing the stationary angular-domain signal essential for subsequent order analysis.

## 3. Numerical Simulation of Unbalance Identification

**Figure 1:**
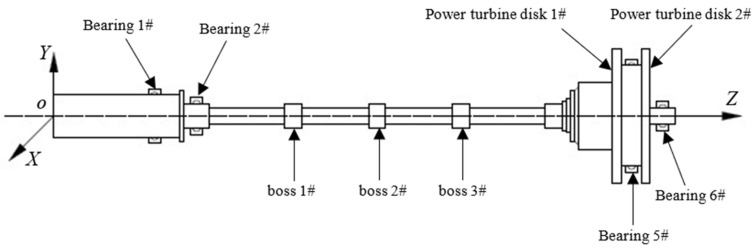
Simulation model of the power turbine rotor.

In the present study, a power turbine rotor was adopted as the numerical simulation model as illustrated in Figure 1. The rotor assembly primarily consists of a power turbine shaft, a first-stage power turbine disk, a second stage power turbine disk and other auxiliary components. The two turbine disks are interconnected via end teeth. Specifically, the power turbine disk 1# is coupled to the power turbine shaft through splines; this connection not only transmits torque but also employs cylindrical centering at both ends for alignment. In contrast, the power turbine disk 2# forms a joint with the shaft using a cylindrical interference fit. The entire rotor features a hollow structural design. Key parameters of the power turbine disks, including their center of mass positions, mass values and moments of inertia, are configured to match those of real world counterparts with high fidelity. The rotor system is supported by four bearings: bearing 1# is a ball bearing, while bearings 2#, 5#, and 7# are roller bearings.

The power turbine rotor is a flexible rotor characterized by a slender, hollow structure and a large aspect ratio. Its specific physical parameters are detailed in Deng [55]. The entire rotor system is evenly divided into 95 segments, corresponding to 96 nodes and 384 degrees of freedom. These 96 nodes are numbered sequentially from node 1 to node 96, starting from the leftmost section (node 1). Given that unbalance is only introduced at boss 1, boss 2, boss 3, and the first-stage power turbine disk of this rotor, the balancing effects at boss 2 and boss 3 are selected as examples to investigate the magnitude of identification differences for unbalance at these four locations.

Based on modal balancing theory, unbalanced excitation forces can be identified using continuous and isolated unbalances, leveraging the principal modes derived from the finite element model of the power turbine rotor system. Accordingly, the first two modes extracted from the rotor’s finite element model are adopted as the principal modes. Following this, unbalance parameters are identified using the computed unbalanced excitation forces.

### 3.1. Numerical Simulation Results for Boss 2

The power turbine rotor operates at a relatively high speed. Therefore, to clearly illustrate the variation in the rotor’s amplitude with rotational speed before and after balancing, the relative rotational speed is defined as

(9) $$\omega_{r}=\frac{\omega (t)}{\omega_{ws}}$$

where $\omega_{ws}$ denotes the rated operating speed of the rotor, and the unit of relative speed is given as a percentage (%).

**Figure 2:**
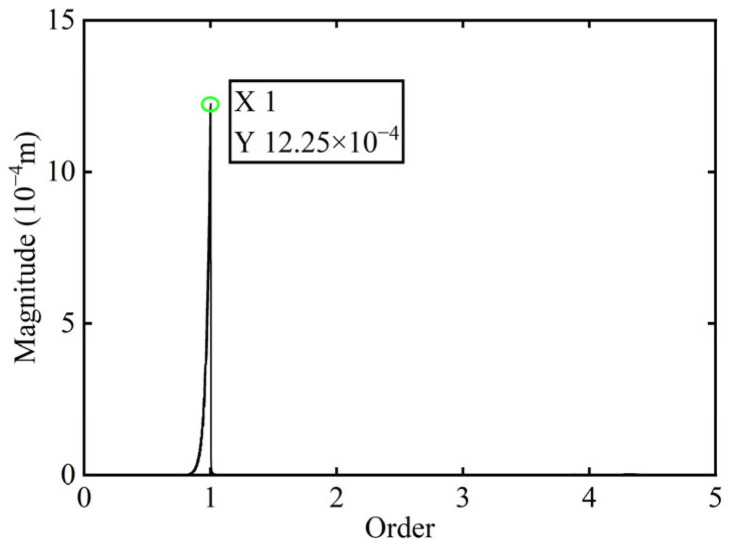
Order spectrum of the transient response signal before balancing of boss 2.

**Figure 3:**
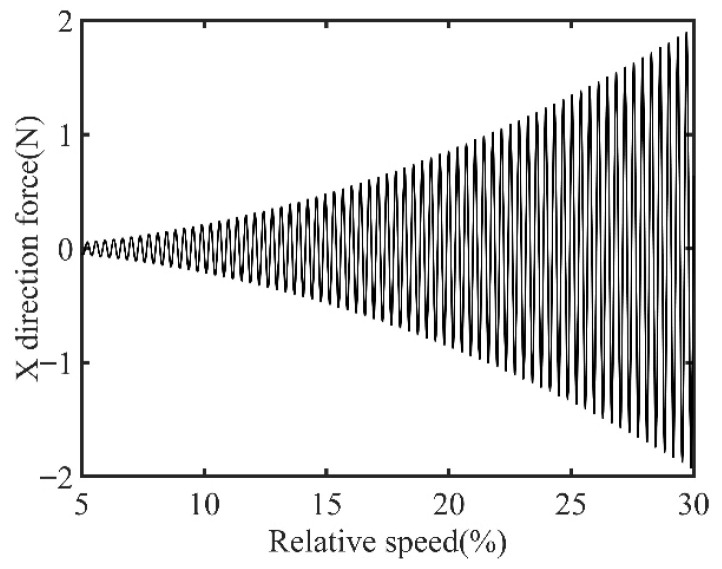
Identified unbalanced excitation forces at boss 2.

First, transient responses are obtained. Subsequently, based on the modal balancing theory applied to boss 2, unbalanced parameters including azimuth, eccentricity, and magnitude are identified using the computed unbalanced excitation forces. The order spectrum of the rotor’s transient response signal before balancing is illustrated in Figure 2 and the unbalanced excitation forces identified at boss 2 of the power turbine rotor by Equation (4) are illustrated in Figure 3.

**Figure 4:**
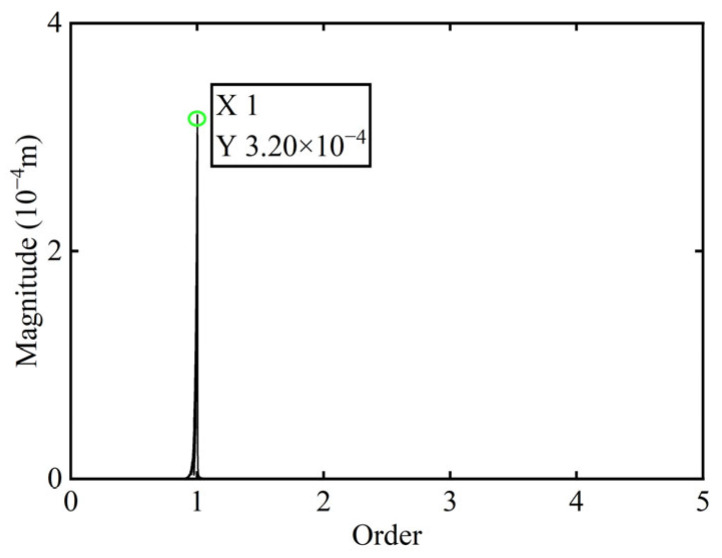
Order spectrum of the transient response signal after balancing of boss 2.

**Figure 5:**
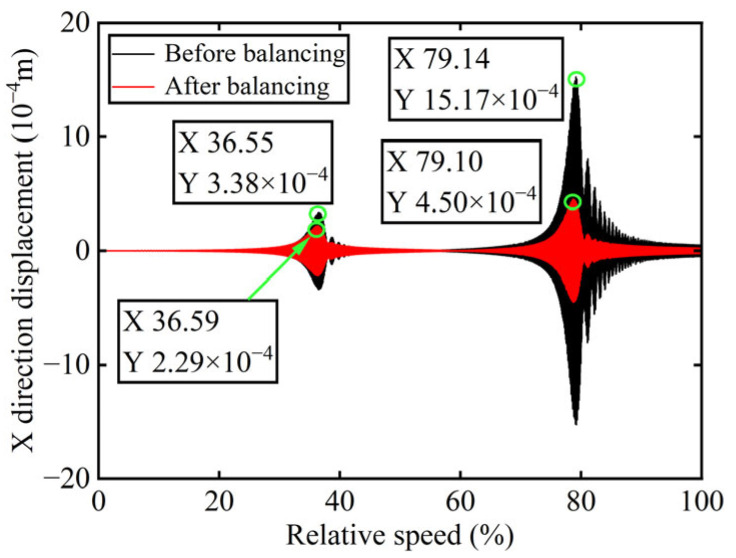
*X*-direction displacement of boss 2 before and after balancing.

**Figure 6:**
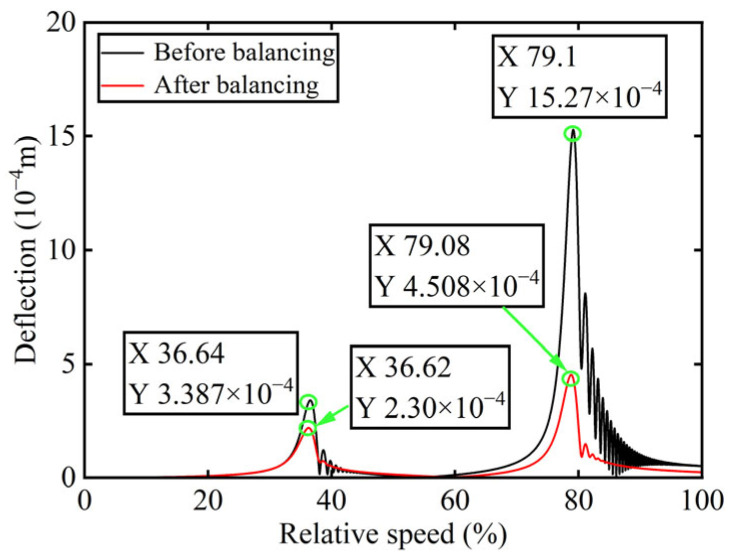
Deflection of boss 2 before and after balancing.

In Figure 3, all minimum values are selected as unbalanced characteristic points to determine the unbalance of the rotor system, enabling the identification of the numerical simulation unbalance value as 0.098 g·mm, and the azimuth of the unbalance is 139.54°. A correction mass of 0.098 g·mm is then added at 319.54° on boss 2. The order spectrum of the rotor’s transient response signal after balancing is illustrated in Figure 4 and the *X*-direction displacement and deflection of the rotor system before and after balancing are presented in Figure 5 and Figure 6, respectively, with the balancing results summarized in Table 1.

As shown in Table 1, the balancing effect of the power turbine rotor is satisfactory after the correction mass is added to boss 2: the amplitude is reduced by 32.09% at the first-order critical speed and by 70.48% at the second-order critical speed, respectively, and as can be seen from Figure 2 and Figure 4, the amplitude at boss 2 decreased by 73.88% after balancing, confirming the accuracy of the unbalance parameter identification.

### 3.2. Numerical Simulation Results for Boss 3

**Figure 7:**
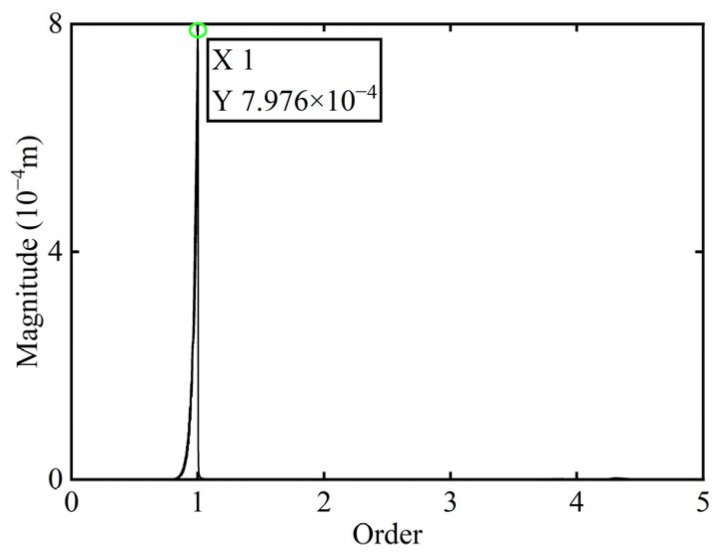
Order spectrum of the transient response signal before balancing of boss 3.

**Figure 8:**
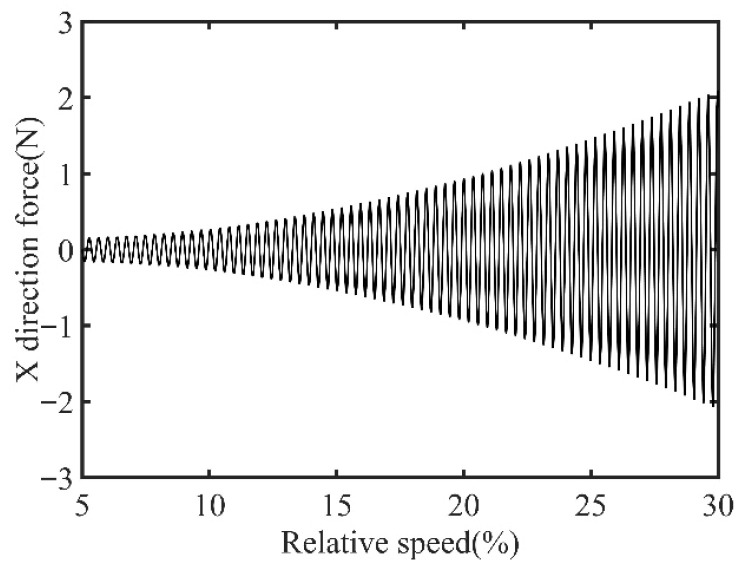
Identified unbalanced excitation forces at boss 3.

Initially, transient responses are acquired. Following this, unbalanced parameters are determined using computed unbalanced excitation forces based on the modal balancing theory implemented for boss 3. The order spectrum of the rotor’s transient response signal before balancing is illustrated in Figure 7. Meanwhile, the unbalanced excitation forces identified at boss 3 of the power turbine rotor by Equation (4) are depicted in Figure 8.

**Figure 9:**
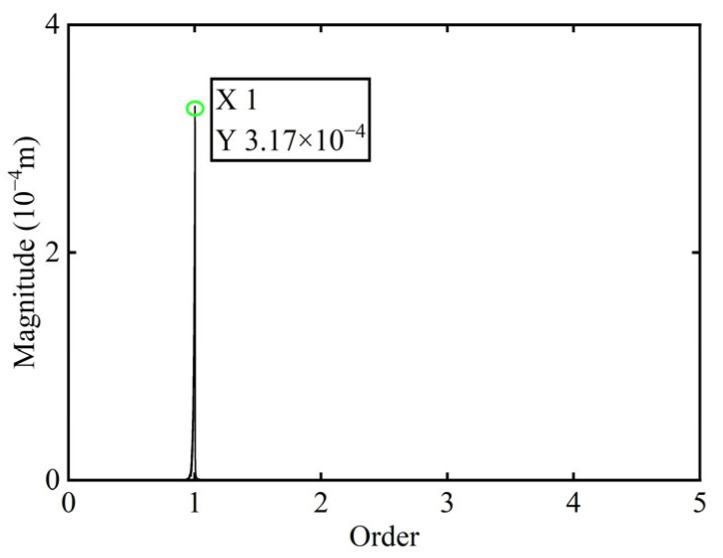
Order spectrum of the transient response signal after balancing of boss 3.

**Figure 10:**
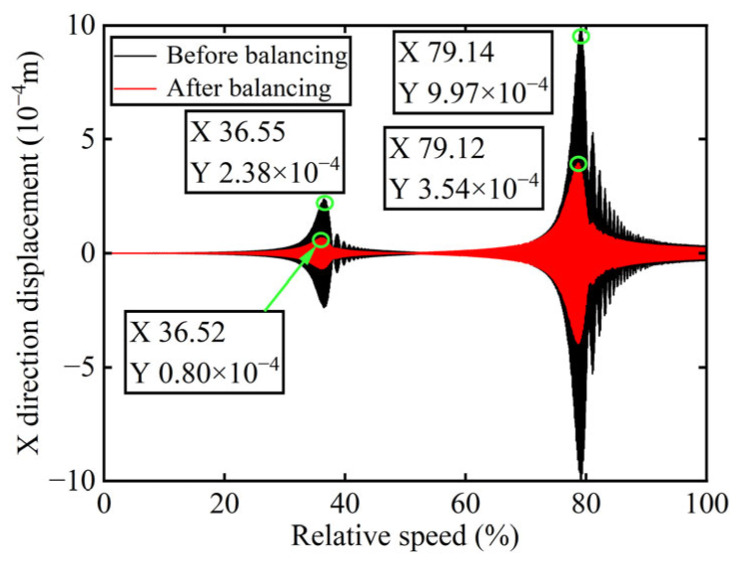
*X*-direction displacement of boss 3 before and after balancing.

**Figure 11:**
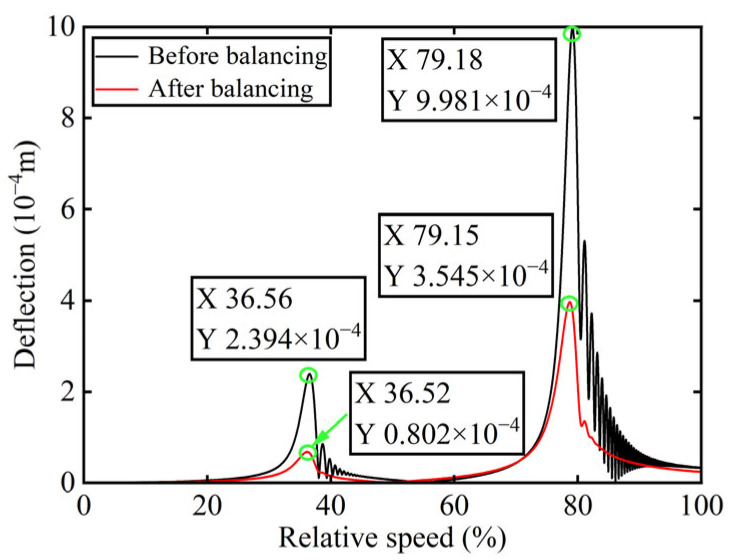
Deflection of boss 3 before and after balancing.

In Figure 8, the unbalance amount can be calculated as 0.103 g·mm, and the azimuth of the unbalance is 145.13°. A balancing mass of 0.103 g·mm is then introduced at 325.13° on boss 3. The order spectrum of the rotor’s transient response signal after balancing is illustrated in Figure 9 and the *X*-direction displacement and deflection of the rotor system prior to and following balancing are depicted in Figure 10 and Figure 11, respectively, with the balancing results summarized in Table 2.

As is evident from Table 2, the power turbine rotor exhibits a favorable balancing effect after the balancing mass is added to boss 3: the amplitude decreases by 66.50% at the first-order critical speed and by 64.48% at the second-order critical speed, respectively, and as shown in Figure 7 and Figure 9, the amplitude at boss 2 decreased by 60.26% after balancing. This demonstrates the accuracy of the parameter identification process.

### 3.3. Comparison of Unbalance Identification Simulation Results Across Four Positions

The unbalanced parameters including azimuth, eccentricity, and mass of boss 1, boss 2, boss 3, and the first-stage power turbine disk in the power turbine rotor system have been identified. The results of unbalance parameter identification at these four locations are presented in Table 3.

As shown in Table 3, the discrepancies between the identified azimuth, mass, and eccentricity values at these four locations on the power turbine rotor fall within ±6°, ±0.12 g, and ±0.15 × 10^−4^ m, respectively. As indicated in the table, the identified unbalances across the four candidate positions on the power turbine rotor demonstrate negligible variation. Therefore, any of these positions is a viable choice for the application of correction masses in both unbalance identification and dynamic balancing procedures.

## 4. Experiment of Unbalance Identification

**Figure 12:**
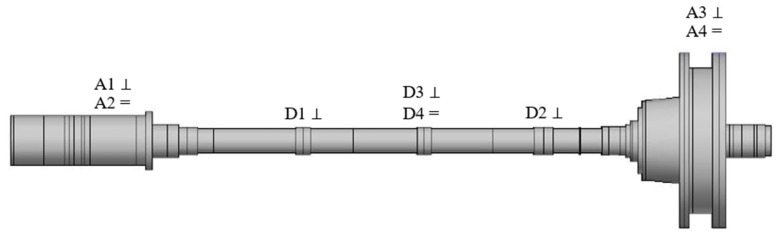
Sensor installation diagram for dynamic balancing experiment of power turbine rotor.

During the transient dynamic balancing without trial weights test, it is necessary to measure the rotor deflection, vibration acceleration of the two supports, and rotational speed. Figure 12 shows the sensor installation diagram for the power turbine rotor dynamic balancing experiment. The rotor deflection is measured by four eddy current displacement sensors (D1~D4), and the vibration acceleration of the two supports that bear the rotor is measured by four acceleration sensors (A1~A4). A photoelectric sensor is used to measure rotational speed. Each boss is fitted with a vertical sensor, while boss 2 is additionally equipped with a horizontal sensor to collect vibration response data of the rotor. Meanwhile, two acceleration sensors are mounted on bearing 2# to monitor rotor vibration and prevent rotor damage in emergency situations.

**Figure 13:**
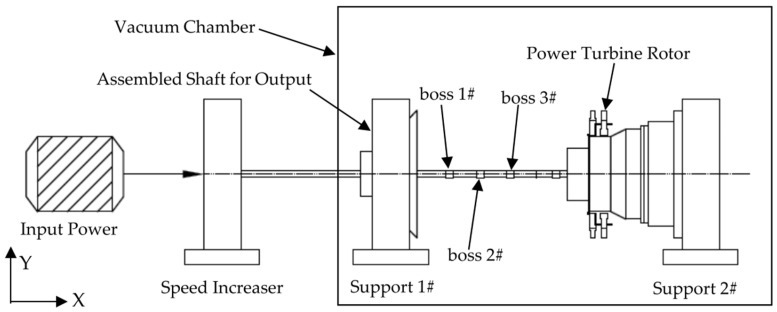
Installation diagram of dynamic balance experiment of power turbine rotor.

The dynamic balancing experiment on the power turbine rotor was performed using a horizontal high-speed rotation test rig. The installation diagram of dynamic balance experiment of power turbine rotor is shown in Figure 13. The test system is made up of a speed regulation system, air pressure control system, power supply system, and real-time monitoring system. A 400 KW DC motor transmits power from the right end of the gearbox shaft through a two-stage speed increaser, and the power turbine rotor is set in rotation by the output shaft assembly. The output shaft assembly utilized in the experiment is in exactly the same condition as that in the actual engine, and it is fastened to the front bracket via an adapter. The rotor test rig is equipped with both high-speed and low-speed ends, as well as a safety enclosure to avoid overloading of the drive motor. Before the experiment, the air pressure control system needs to be vacuumed. During the startup phase, the rotational speed was managed by professional operators, with an average acceleration of around 13 rad/s^2^.

Consistent with the numerical simulation, the experiment involved conducting unbalance identification and adding correction masses on boss 2 and boss 3 of the power turbine rotor, with the balancing effect observed. Following this, the unbalance identification results for boss 1, boss 2, boss 3, and the first-stage power turbine disk are compiled and compared.

### 4.1. Experimental Results for Boss 2

**Figure 14:**
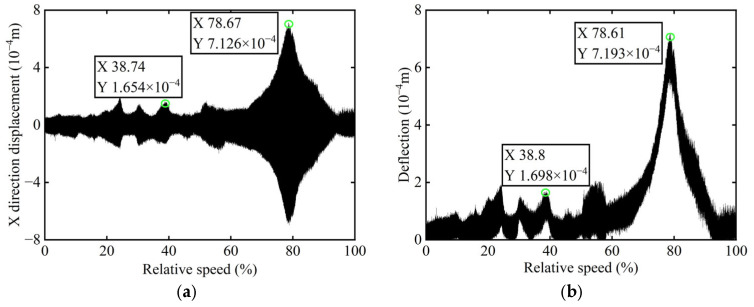
Transient vibration response before balancing of boss 2: (**a**) X-direction displacement; (**b**) Deflection.

The original transient vibration response of the *X*-direction and the deflection before balancing of boss 2 of the rotor are shown in Figure 14.

**Figure 15:**
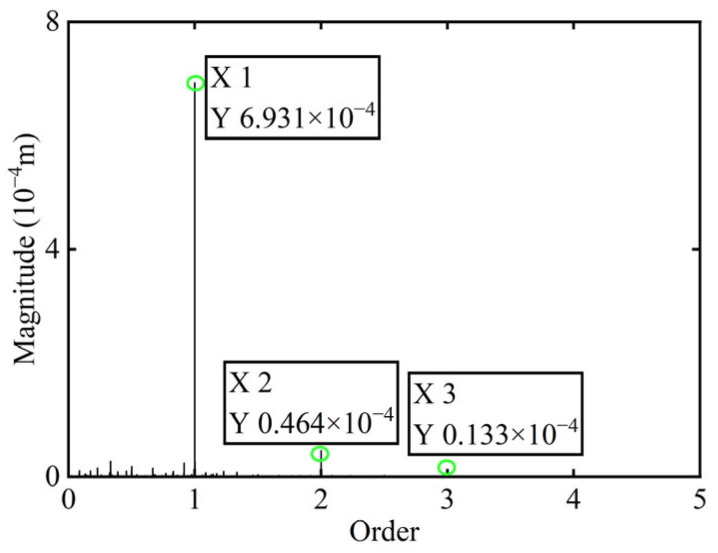
Order spectrum before balancing of boss 2.

In Figure 14, it is observed that the original transient vibration response and deflection fail to clearly exhibit their change characteristics, which can be attributed to experiment conditions and other interfering factors. Consequently, directly identifying unbalance using the collected signals would significantly compromise the accuracy of the identification process. Therefore, order analysis is required for the acquired signals in order to remove non-negligible background noise and several additional frequency components, thereby allowing for the extraction of the 1X component for further analysis. The order spectrum before balancing of boss 2 is illustrated in Figure 15.

**Figure 16:**
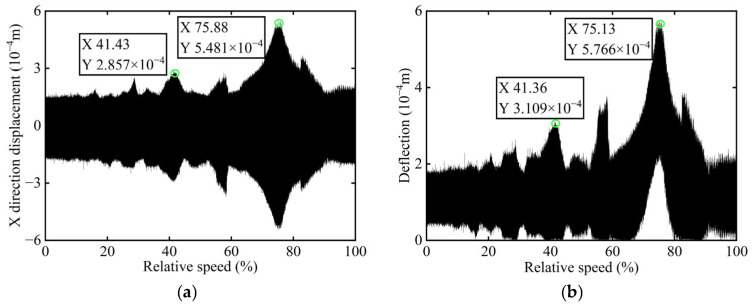
Transient vibration response after balancing of boss 2: (**a**) *X*-direction displacement; (**b**) Deflection.

**Figure 17:**
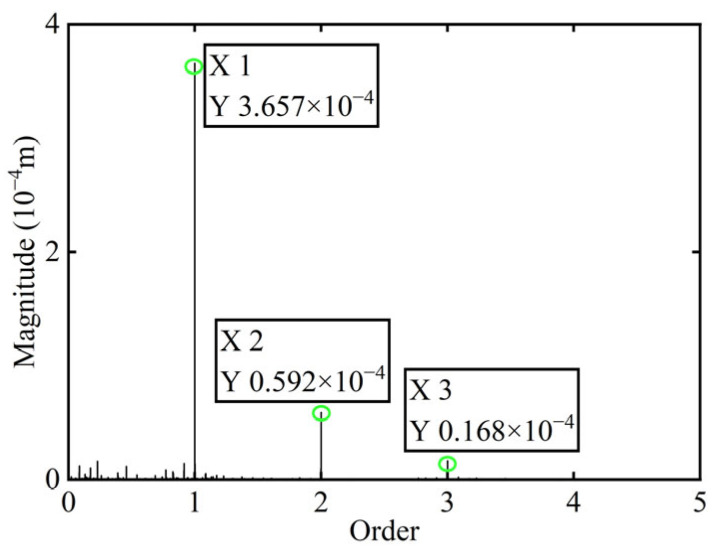
Order spectrum after balancing of boss 2.

The unbalance of the rotor was identified using the method proposed in this study. The calculated unbalance parameters yield a result of 0.049 g·mm, and the azimuth of the unbalance is 147.94°. After a correction mass of 0.049 g·mm was added at 327.94° on boss 2 and the power turbine rotor was restarted, the *X*-direction transient vibration response and deflection and the order spectrum of boss 2 after balancing in the power turbine rotor are presented in Figure 16 and Figure 17, respectively.

**Figure 18:**
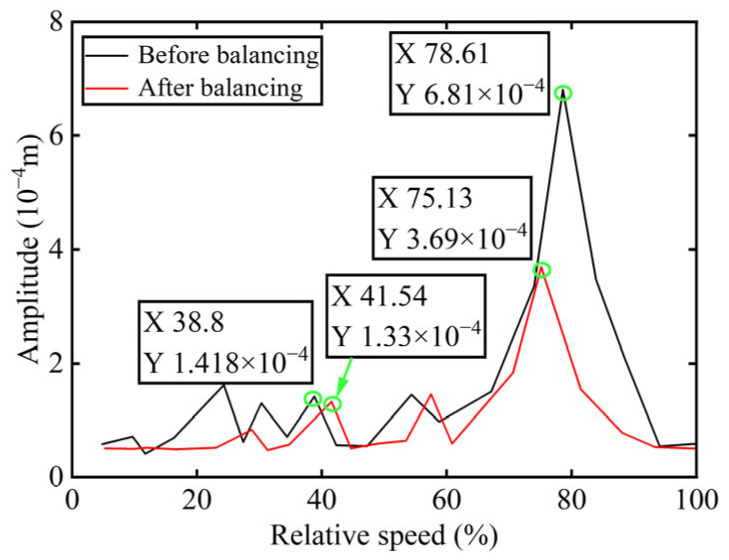
The amplitude diagram of boss 2 before and after balancing.

Bode diagrams calculated before and after balancing are commonly used to analyze balancing efficiency more intuitively. Accordingly, the amplitude diagram of the power turbine rotor before and after balancing is presented in Figure 18, with the balancing results summarized in Table 4.

As indicated in Table 4, the power turbine rotor demonstrates an effective balancing outcome following the addition of correction mass to boss 2. Specifically, the amplitude at the first-order critical speed is reduced by 6.21%, while at the second-order critical speed, the reduction reaches 45.81%. And as shown in Figure 15 and Figure 17, the amplitude of the 1X component at boss 2 declined by 47.24% after balancing. These results serve to confirm the precision of identifying unbalance parameters.

### 4.2. Experimental Results for Boss 3

**Figure 19:**
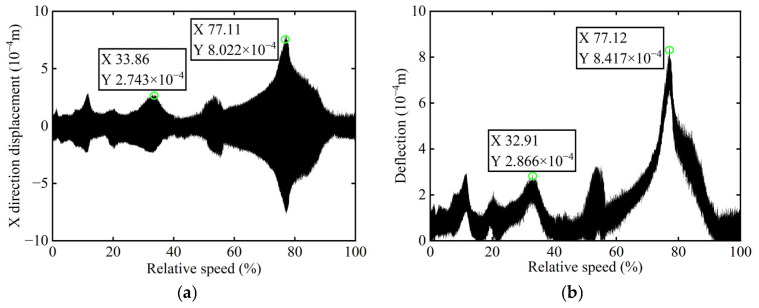
Transient vibration response before balancing of boss 3: (**a**) *X*-direction displacement; (**b**) Deflection.

**Figure 20:**
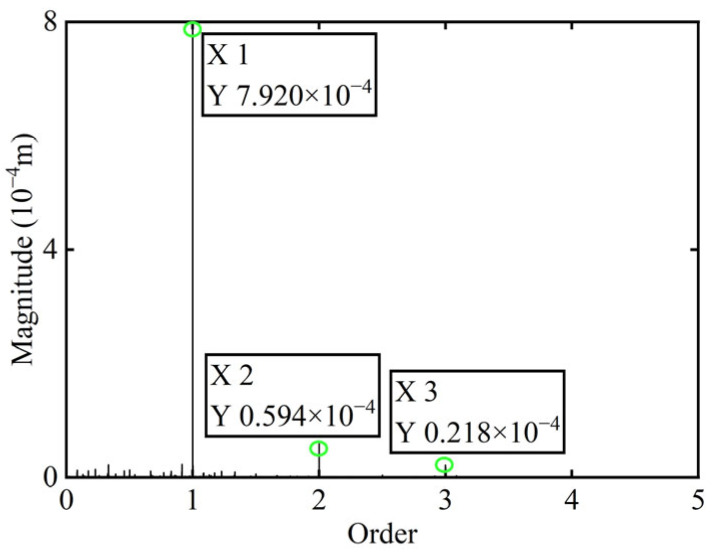
Order spectrum before balancing of boss 3.

The original transient vibration response of the *X*-direction and the deflection before balancing of boss 3 of the rotor are shown in Figure 19. The original transient response signals collected were subjected to order analysis. The 1X component was then extracted for further analysis, and the order spectrum before balancing of boss 3 is illustrated in Figure 20.

**Figure 21:**
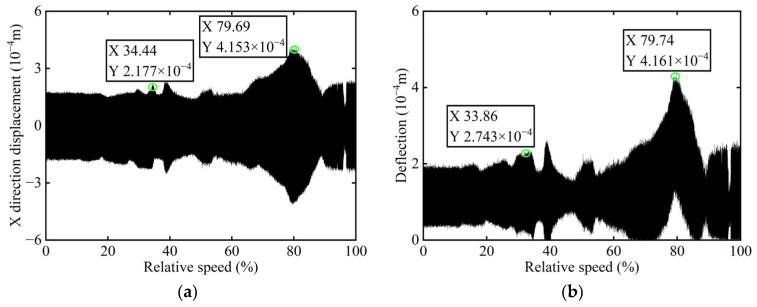
Transient vibration response after balancing of boss 3: (**a**) *X*-direction displacement; (**b**) Deflection.

**Figure 22:**
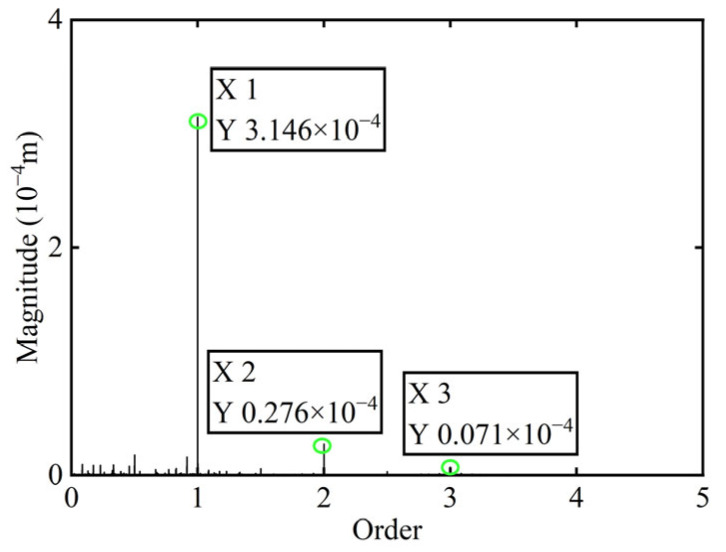
Order spectrum after balancing of boss 3.

The computed unbalance parameters yield a result of 0.055 g·mm, and the azimuth of the unbalance is 152.13°. Following the addition of a correction mass of 0.055 g·mm at 332.13° on boss 3 and the subsequent restart of the power turbine rotor, the *X*-direction transient vibration response and deflection and the order spectrum of the power turbine rotor’s boss 3 after balancing are illustrated in Figure 21 and Figure 22, respectively.

**Figure 23:**
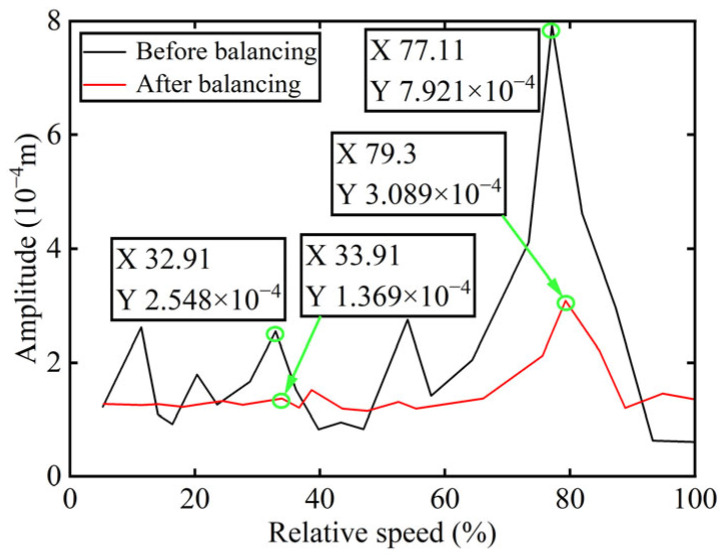
The amplitude diagram of boss 3 before and after balancing.

The amplitude diagram of the power turbine rotor before and after balancing is presented in Figure 23, with the balancing results summarized in Table 5.

As seen in Table 5, the power turbine rotor achieves a favorable balancing effect after the correction mass is added to boss 3. Specifically, the amplitude at the first-order critical speed is reduced by 46.27%, and at the second-order critical speed, the reduction reaches 61.00%. And as shown in Figure 20 and Figure 22, the amplitude of 1X component at boss 2 declined by 60.28% after balancing. These findings validate the precision of identifying unbalance parameters.

### 4.3. Comparison of Unbalance Identification Experiment Results Across Four Positions

Unbalance parameters including azimuth, eccentricity and mass were identified for boss 1, boss 2, boss 3 and the first-stage power turbine disk within the power turbine rotor system. The results of unbalance parameter identification at these four positions are compiled in Table 6.

As indicated in Table 6, the discrepancies in identified azimuth, mass, and eccentricity among the four positions on the power turbine rotor fall within ±5°, ±0.11 g, and ±0.10 × 10^−4^ m, respectively. The results presented in the table show minimal discrepancy in the identified unbalances across the four correction positions, thereby validating the findings from the numerical simulation. Consequently, any of these positions can be effectively employed as a correction plane for unbalance identification and dynamic balancing.

## 5. Discussion

Through numerical simulations and experimental studies on the power turbine rotor of an aero-engine, this paper not only verifies the applicability of the proposed transient balancing without trial weights method but also confirms that rotor dynamic characteristics can be measured and unbalances identified at any axial correction mass position. This facilitates on-site dynamic balancing operations, enabling unbalance identification at any location within limited spatial constraints during on-site balancing processes and laying a prerequisite for subsequent research on on-site dynamic balancing.

## 6. Conclusions

In this paper, the effectiveness of dynamic balancing without trial weights is investigated through both numerical simulation and experiments on the power turbine rotor. Additionally, the principle of the order analysis is introduced. Furthermore, the proposed dynamic balancing method is employed to determine unbalance parameters at boss 1, boss 2, boss 3, and the first-stage power turbine disk, respectively, with the differences among these four positions compared. The main conclusions are as follows:

1. Digital signal processing significantly improves the identification accuracy of unbalance without altering the amplitude or location of the critical speeds, while the identification accuracy of unbalance in the power turbine rotor is notably improved.
2. Using the dynamic balancing method proposed in this study, numerical simulations and experiments were performed on boss 2 and boss 3. The findings indicate that the amplitude at the critical speed is notably reduced, with a more pronounced effect observed at the second-order critical speed.
3. Numerical simulations and experimental comparisons were carried out on the identified unbalances at boss 1, boss 2, boss 3, and the first-stage power turbine disk of the power turbine rotor. The results show that the unbalance identification is consistent across all four positions, with negligible discrepancies.

## Figures and Tables

**Table 1:** Numerical simulation balancing results of boss 2.

Critical Speed	Before Balancing /(10^−4^ m)	After Balancing /(10^−4^ m)	Reduction in Vibration Amplitude/%
First-order	3.387	2.3	32.09
Second-order	15.27	4.508	70.48

**Table 2:** Numerical simulation balancing results of boss 3.

Critical Speed	Before Balancing /(10^−4^ m)	After Balancing /(10^−4^ m)	Reduction in Vibration Amplitude/%
First-order	2.394	0.802	66.50
Second-order	9.981	3.545	64.48

**Table 3:** Comparison of numerical simulation results for unbalances across different positions.

Measured Position	Azimuth /(°)	Weight /(g)	Eccentricity /(10^−4^ m)
boss 1#	143.94	0.73	1.52
boss 2#	139.54	0.65	1.51
boss 3#	145.13	0.69	1.49
Disk 1#	140.22	0.61	1.64

**Table 4:** Experimental balancing results of boss 2.

Critical Speed	Before Balancing /(10^−4^ m)	After Balancing /(10^−4^ m)	Reduction in Vibration Amplitude/%
First-order	1.418	1.33	6.21
Second-order	6.81	3.69	45.81

**Table 5:** Experimental balancing results of boss 3.

Critical Speed	Before Balancing /(10^−4^ m)	After Balancing /(10^−4^ m)	Reduction in Vibration Amplitude/%
First-order	2.548	1.369	46.27
Second-order	7.921	3.089	61.00

**Table 6:** Comparison of experiment results for unbalances across different positions.

Measured Position	Azimuth /(°)	Weight /(g)	Eccentricity /(10^−4^ m)
boss 1#	150.75	0.49	1.40
boss 2#	147.94	0.38	1.30
boss 3#	152.13	0.42	1.32
Disk 1#	151.46	0.45	1.35

## Data Availability

Data are contained within the article.

## Nomenclature

$\phi_{n}(z)$	*n*th principal mode
$S_{n}$	Modal mass of the *n*th mode
m(z)	Continuous mass along the *z*-axis
*l*	Total length of the shaft
P(z)	Distribution function of rotor unbalance
$P_{s}$	Unbalance equivalent of the *s*th-order mode shape.
$U_{K}$	Isolated unbalance at point *K*
$U_{p}$	Continuous unbalance of the rotor system
$q_{n}$	*n*th-order modal component of the unbalanced distribution
ω(t)	Angular velocity of the power turbine rotor
$m_{r}$	Mass of the *r*-th disk
$e_{r}$	Eccentricity of the *r*-th disk
$\theta_{ur}$	Initial unbalance azimuth of the *r*-th disk
a(t)	Angular acceleration of the power turbine rotor
α(t)	Phase term
κ(t)	Rotational angle of the disk
$\omega_{ws}$	Rated operating speed of the rotor
*O*	Order
*f*	Frequency
*n_rs_*	Rotating speed
